# Robot-assisted sleeve gastrectomy in patients with obesity with a novel Chinese domestic MicroHand SII surgical system

**DOI:** 10.1186/s12893-021-01259-3

**Published:** 2021-05-25

**Authors:** Weizheng Li, Kang Kong, Pengzhou Li, Guohui Wang, Beibei Cui, Liyong Zhu, Shaihong Zhu

**Affiliations:** 1 Department of General Surgery, Third Xiangya Hospital, Central South University, No.138 Tongzipo Road Yuelu District, Changsha, 410013 Hunan People’s Republic of China; 2 Key Laboratory of Mechanism Theory and Equipment Design of Ministry of Education, Tianjin University, No. 92 Weijin Road Nankai District, Tianjin, 300354 People’s Republic of China

**Keywords:** Robot-assisted surgery, Sleeve gastrectomy, Obesity

## Abstract

**Background:**

A new device has been added to the Chinese MicroHand surgical robot family, developed based on the successful application of control algorithms. As a benefit of using these specialized control algorithms, the motion mapping relation can be accommodated into the system without the help of a built-in image system, resulting in a novel Chinese domestic surgical robot with two arms called MicroHand SII, which is different from the former MicroHand S and da Vinci systems. In this study, we investigate the performance of a novel MicroHand SII robotic platform in patients with obesity.

**Methods:**

From March 2018 to April 2019, a total of 7 patients whose BMI ranged from 29.9 to 49.8 kg/m^2^ were operated on with the robot-assisted technique using the MicroHand SII surgical system. Data regarding demography, surgical procedure and the 3-month outcome postoperation were collected.

**Results:**

There were 2 female and 5 male patients with a median age (range) of 35 (20–51) years. The median operative time was 160 (149–195) minutes. None were converted to open surgery. All anthropometry indices improved significantly (p < 0.05) at 3 months postoperatively. There were no cases of surgical site infection, gastrointestinal/abdominal bleeding, or conversion to an open operation.

**Conclusions:**

The initial experience showed that the Chinese domestic robot surgical system MicroHand SII could be feasibly and safely applied in sleeve gastrectomy in patients with obesity. Because of the unique design of this system such as a two-hand robot, an array of master–slave motion strategies, and a roll joint at the end of the instruments that allows 7 degrees of freedom, this robotic platform has presented its own obvious advantages.

## Background

Obesity has growing prevalence worldwide, causing a series of severe problems in public health [1]. Compared to non-surgical interventions, bariatric surgery can dramatically reduce body weight in a lasting manner and ameliorate comorbidities in patients with obesity [2, 3]. At present, hundreds of thousands of patients with obesity receive bariatric surgery every year [4]. Laparoscopic sleeve gastrectomy (LSG) has become the most common procedure in bariatric surgery according to an estimation of the International Federation for the Surgery of Obesity and Metabolic Disorders (IFSO) [5]. However, it is difficult to dissect and mobilize a deep-lying gastric fundus in many of these patients with morbid obesity using the typical laparoscopic platforms currently available.

With the development of robotic surgical systems, robotic surgery has appeared as a new category of minimally invasive surgery [6]. Robotic technology with multiple degrees of freedom (DoF) and tridimensional (3D) imaging can improve operating dexterity, visualization of difficult anatomic locations, and hand–eye coordination in surgery. The robot surgical system compensates for the technical limitations of laparoscopic instruments and solves many problems caused by human restrictions, such as fatigue and low precision.

The robotic platform has gained widespread utilization in bariatric surgery for the treatment of morbid obesity in recent years [7]. The Metabolic and Bariatric Surgery Accreditation and Quality Improvement Program (MBSAQIP) database showed that, in the last few years, the da Vinci robotic platform has increasingly emerged as an attractive technology in bariatric surgery [8, 9]. At present, da Vinci sleeve gastrectomy has been carried out, showing good results [10, 11]. Robotic-assisted sleeve gastrectomy offers a viable platform to surgeons performing sleeve gastrectomy.

However, the high costs of the da Vinci robotic system make it very difficult to routinely apply it to bariatric surgery [11, 12]. Moreover, its expensive annual maintenance requirements and surgical consumables limit the application of the da Vinci surgical system in China. However, recently, the Chinese domestic MicroHand surgical system was developed with a compact structure and low cost. Similar to the da Vinci surgical robot, the MicroHand series surgical robot is a kind of master–slave robot [13].

Recently, the MicroHand surgical system added a novel robot called the MicroHand SII Chinese domestic surgical robot. The MicroHand SII system was developed in 2015 by two universities. We have witnessed the performance of the MicroHand S robotic platform developed in 2013 in primary clinical applications. According to previous studies [14, 15], MicroHand S has been applied to appendectomy, cholecystectomy, and right hemicolectomy, without deep organs involved. Therefore, we are highly interested in a novel two-arm robot used in deep organs. The purpose of this study is to investigate the performance of the MicroHand SII robotic platform by accomplishing the surgical task analysis of sleeve gastrectomy in patients with obesity.

## Methods

Seven patients with obesity who met the metabolic surgery criteria set by the Chinese Society for Metabolic & Bariatric Surgery (CSMBS) underwent MicroHand SII robot-assisted sleeve gastrectomy (MIISG) from March 2018 to April 2019. All patients suffered from fatty liver with hypertrophied hepatic lobes. Perioperative overall operative time, preoperative setup time, intraoperative blood loss, hospital stays, and perioperative complications were recorded. Anthropometry data, including body weight, body mass index (BMI) and waist circumference (WC) preoperatively and 3 months postoperatively were collected. Percentage excess weight loss (% EWL) was estimated in the following 3 months. % EWL = (weight loss/baseline excess weight) × 100%, where baseline excess weight = baseline weight − ideal weight. The ideal weight is based on a person’s weight at a BMI of 25 kg/m^2^. All results were expressed as a mean ± standard deviation. The paired-samples T test was used, and the significance level was set at a two-tailed α = 0.05. The surgeons had sufficient training to adapt well to the MicroHand SII robot. Written informed consent was obtained from each patient. This study was authorized by the ethics committee of the hospital and registered in the National Institutes of Health website: www.clincaltrials.gov. The registration identifier is NCT02752698.

### Overview of the MicroHand SII robot surgical system

**Fig. 1:**
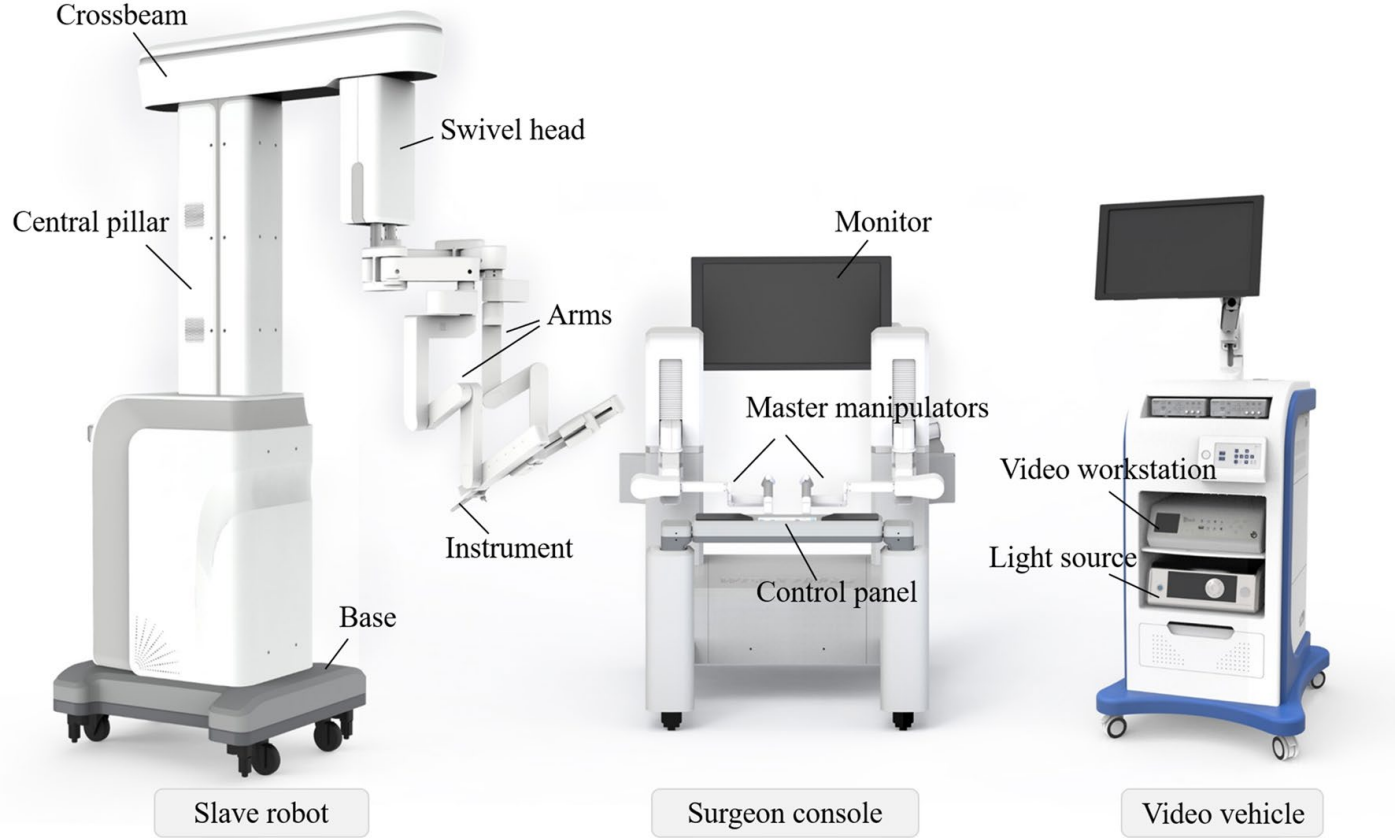
The structure of MicroHand SII robot. The system consists of a surgeon console (middle), a slave robot (left) and a video vehicle (right). An external monitor, two master manipulators and a control panel are integrated into the surgeon console. The slave robot is designed for a hoisting beam structure, which is composed of a base, a central pillar, a crossbeam, a swivel head, two arms and multi-DoF instruments. The video vehicle consists of a video workstation and light source. The central pillar is configured in the base, which can rise and fall in the vertical direction. The crossbeam is installed on the central pillar, which can translate along the horizontal axis of the pillar. A swivel head is mounted on the other end of the crossbeam. The swivel head can rotate around the vertical axis in two directions with a range of motion of [− 90°, 90°]

The MicroHand SII robot surgical system mainly consists of a surgeon console, a slave robot and a video vehicle (Fig. 1), the structure and appearance of which are distinctly different from those of the da Vinci surgical system. The preloaded cable-driven system and harmonic reducers make it light in weight, compact in structure, and enable a large range of motion. Compared to the MicroHand S, this robot was upgraded successfully in configuration, such as being equipped with an ultrasonic scalpel, a master–slave motion scaling function and an audible alarm function. Both the scaling and alarm functions are integrated into the control panel for quick manual manipulation. Benefitting from using specialized control algorithms, the motion mapping relation can be accommodated into the system without the help of a built-in image system, which are different from the MicroHand S and da Vinci systems. Therefore, the two-arm MicroHand SII is compatible with conventional endoscopic image systems in hospitals, in which the manipulator used to hold the endoscope is abolished. Thanks to this design, the cost, volume and weight of the MicroHand SII can be further reduced.

**Fig. 2:**
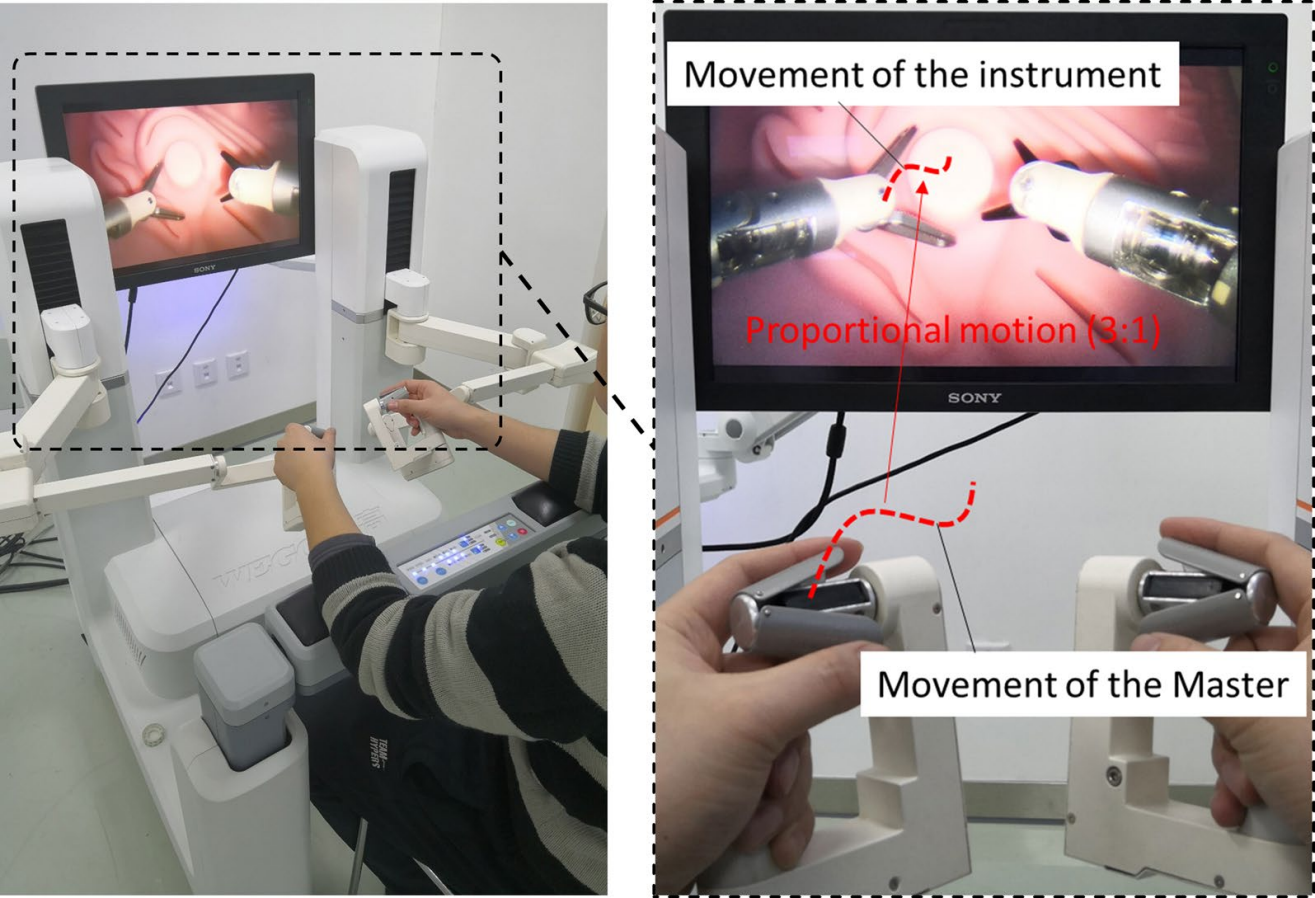
The control of MicroHand SII. The surgeon holds the joystick, with the thumb and index finger holding the clip and the other fingers pressing the trigger on the handle, to start the instrument, and the surgeon controls the movement of the joints of the handle to realize the corresponding actions of the instrument

The surgeon console allows the surgeon to control multi-DoF instruments by operating master manipulators and foot pedals. The two master manipulators conform to the ergonomic engineering facilitating to filter out hand tremors, relieve hand fatigue and improve precision. Owing to the embedded controller, the motions input by operating master manipulators outside the abdomen can be mapped to the end-effector motions of instruments inside the abdomen cavity exactly. The reproduced motions of the instruments following the master manipulators are activated by grasping the button on the handle. Then, incremental motion is used to reposition the master manipulators during surgery to solve the mutual interference or motion limits of the master manipulators, which is implemented by the clutch mechanism fired by pinching the clamp to disengage the instrument motion from the corresponding motion of the master manipulators. The control panel is used for system initialization and to establish the initial values of certain key parameters prior to surgery. The motion scale between the master and the slave is adjustable among different proportions, such as 3:1, 6:1 and 10:1, by simply clicking the keys on the control panel at any time during the entire procedure (as shown in Fig. 2).

In order to meet the requirement of precise operation, a 3:1 proportional motion control is introduced. The 3:1 proportional motion control means that the range of motion at the end of the instrument is 1/3 of that of the surgeon's master hand. For different operating region, surgeon can choose different proportional motion control. For example, when transecting the gastrocolic ligament from the greater curvature of the stomach using the ultrasonic scalpel, the surgeon usually chooses 3:1 to complete resection quickly. When isolating the short gastric vessel, the surgeon can choose 10:1 to refine the operation. The master–slave motion mapping strategies of MicroHand SII are a unique design. The setting can be turned up in fine operations and turned down in extensive operations. This design can improve the safety and speed up the process of the operation and has been well applied in operations.

**Fig. 3:**
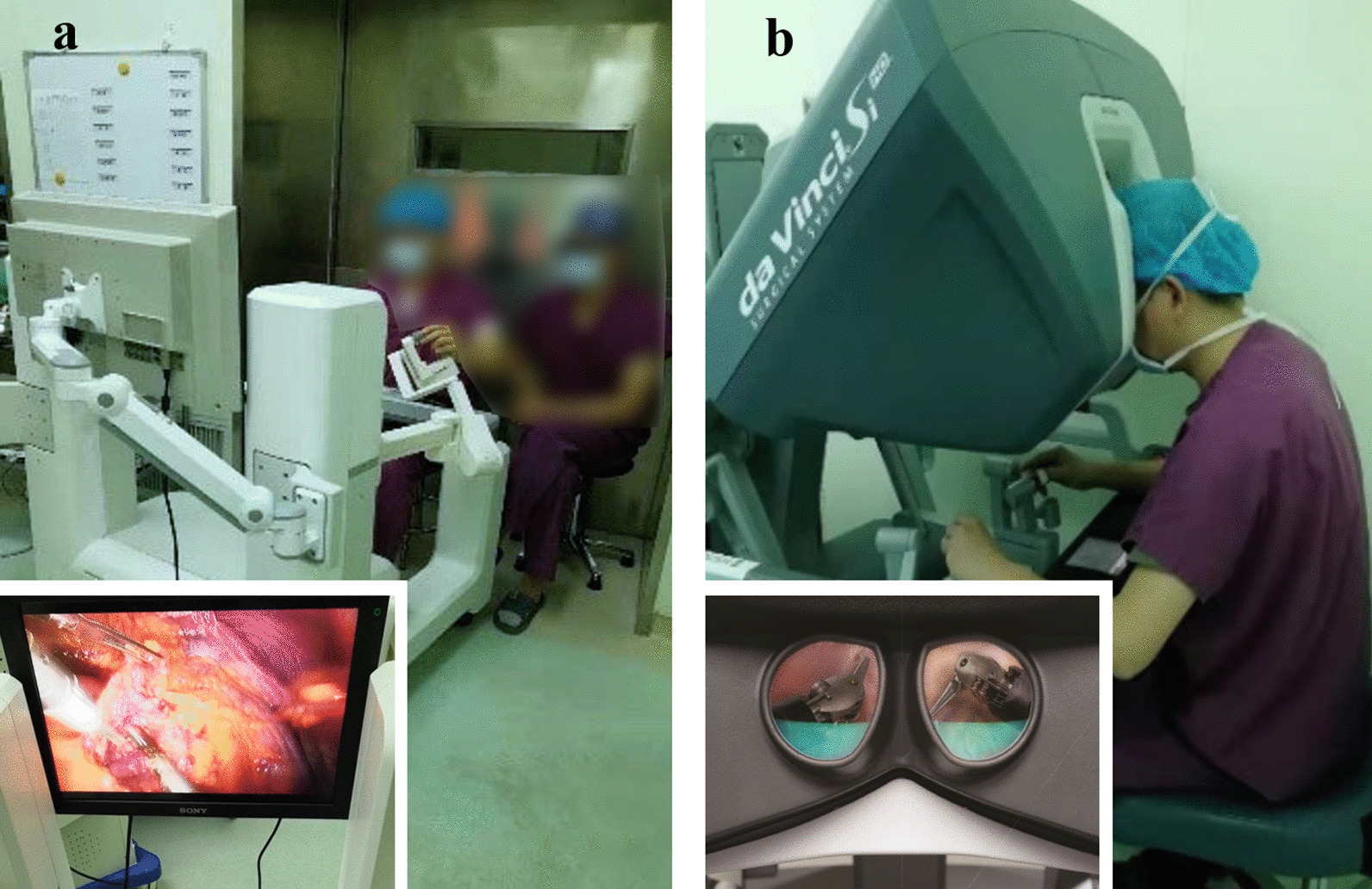
**a** The MicroHand SII system with an open-field viewer. **b** The da Vinci system with a closed image viewer

The audible alarm function is attributed to the sensor equipped in each passive joint, which is to guarantee operation safety. The control system compares the angular positions provided by the sensors and those obtained through the kinematic calculation at each controller time-step. Once the error between them exceeds a certain range, the robot is stopped immediately, and an alarm sounds in the control panel. The surgeon needs clear out the fault first by clicking the button on the control panel and then continue the surgical procedure. The imaging system transmits a stable 3D view in an open-field way (Fig. 3a). Benefitting from the open high-definition 3D view of MicroHand SII, an easier real-time discussion and teaching intraoperative is allowed for, which is inconvenient with a closed image viewer integrated in the console in da Vinci (Fig. 3b). In addition, it is helpful to relieve the surgeon’s neck fatigue without persistently laying the head down against the image viewer for a long time.

**Fig. 4:**
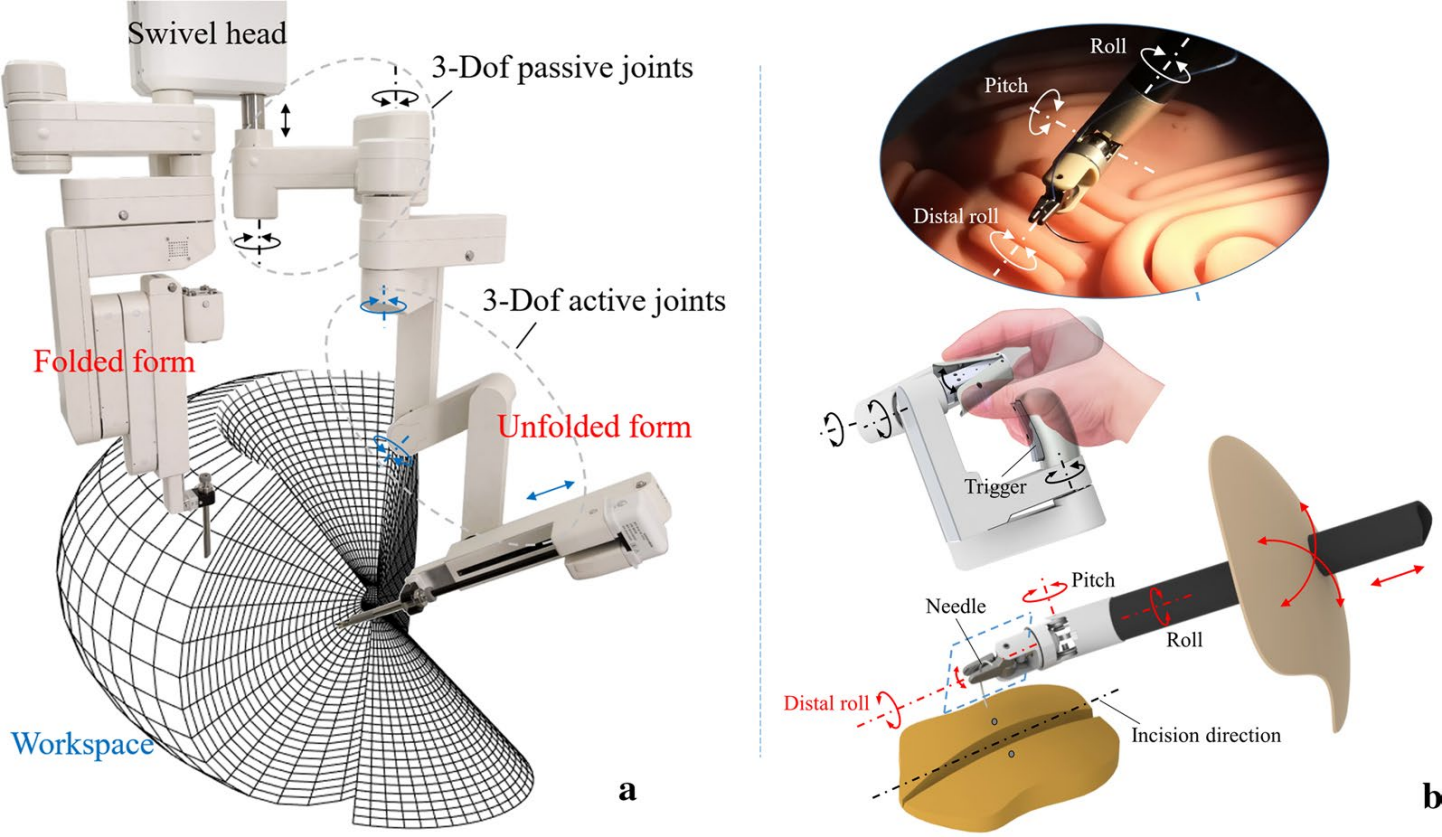
**a** The introduction of a robotic arm to MicroHand SII. The robotic arm has three active joints and three passive joints in each slave manipulator. The compact size and light weight of the operating arm are accomplished by adopting the fold-unfold structure design, which can provide a large-scale moving area. The two arms can be located in the same plane after their joints collapse tightly. The instrument can move 180° in the left-to-right direction and 180° in the foot-to-head direction to reach the full extent of the abdomen. The design of the active slave manipulator combined with the central pillar, crossbeam, and long-range swivel head structure can fulfil the surgical workspace of its instruments. **b** The multi-DoF instruments. The joint motion of the handle controls the movement of the end of the instrument, while the motion of the main hand arm controls the movement of the instrument

The operating arm system is optimized with two movable manipulators installed on the swivel head used to place the instruments (Fig. 4a). The structure of the instrument is designed optimally on the basis of the kinematic analysis together with the ergonomic index. To ensure that the surgical robot can perform the suture motion, which is largely a rotation about the bisector line of the two jaws of the instrument, an end rotational motion needs to be realized. Based on this, a separated roll joint is designed at the distal end of the instrument (Fig. 4b). Eventually, the system allows the manipulator to move with seven DoFs beyond the laparoscopic surgery, technically.

### The technical advantages in patient–robot interaction

Patients with obesity have inherent surgical features that greatly increase the operation difficulty. During MIISG, the target organ is the stomach in the upper abdominal region; the robot is used to assist in operations. In terms of the LSG surgical requirements, problems related to thick abdominal walls, a mass of visceral fat, deep stomach fundus and limited space to manoeuvre the instrument are always encountered. At the same time, the anterior and posterior diameters of the abdominal cavity are large, and stomach fundus and part of the gastric body in patients with obesity are covered by the hypertrophied fatty left lobe of liver. However, MicroHand SII has some particular features, especially in patient–robot interaction, to overcome the difficulties in exposure and operation.

It is convenient to locate the slave robot owing to its crossbeam design and flexible swivel head. The route of movement up and down the column of the slave robot is long-range, which is enough to overcome the elevated bed after patients with obesity lay on the bed. The surgeon need not intentionally turn down the bed to adapt to the slave robot. This design can free up space around the patient’s head, which makes general anaesthesia easier during a robotic surgery. It is beneficial to place and settle surgical equipment, including the anaesthetist cabinet, several sterile trays, display monitors, several instrument cupboards, and so on, to satisfy operation room area requirements. However, many pieces of equipment are always stored in every area until needed for surgery. This compact robot does not occupy much room around the patient, keeping the surgeon or assistants close to the patient.

During the whole intervention of the MIISG, benefitting from its optimal design, although patients with obesity are large, we can always place the slave robot acceptably without repeated adjustments. The slave robot of the MicroHand SII is well-suited to this situation and can be placed without the need to move and dock the robotic cart several times. It is not affected by the position of the operating bed only meeting the basic placement principle, which is simply that the slave robot is near to the lesion side. The operation site can be completely covered by the workspace of the robot to avoid the need to reposition the robot during the surgery. In the da Vinci surgical system, the slave robot must be placed at a special site near the operation bed. Therefore, the position of the surgical cart is simpler than in the da Vinci system, without repositioning during the entire procedure.

The two manipulators of the slave cart have long arms, which are suited for long-scale adjustments. The kinematic design of the robot arm can avoid the incision point constraint as is present with conventional laparoscopy which means the point of insertion of the instrument in the abdominal wall is fixed, thereby constraining various motions of the non-jointed instruments [16]. The number of joints is set reasonably, and the length proportion of each arm is set appropriately. In the contracted state, each arm is folded together, occupying little space. In the open state, each arm is fully and freely extended to generate a very wide operating field for encircling, avoiding external mechanical interference between the arms. These designs not only meet the space requirements of the abdominal operating area but also meet the requirements of the operating triangle of the working instruments. Therefore, it is easy to position the surgical cart and trocars. The robot can release some of the space around the operating table that may be occupied by the robot arms compared with the da Vinci assisted system. The 450 mm length of the instruments, including robotic graspers, needle holder and ultrasonic scalpel, is longer than that of the da Vinci system, which can fulfil the motion requirements with a maximum moving range of 250 mm for the back and forth motion measured from the incision point.

**Fig. 5:**
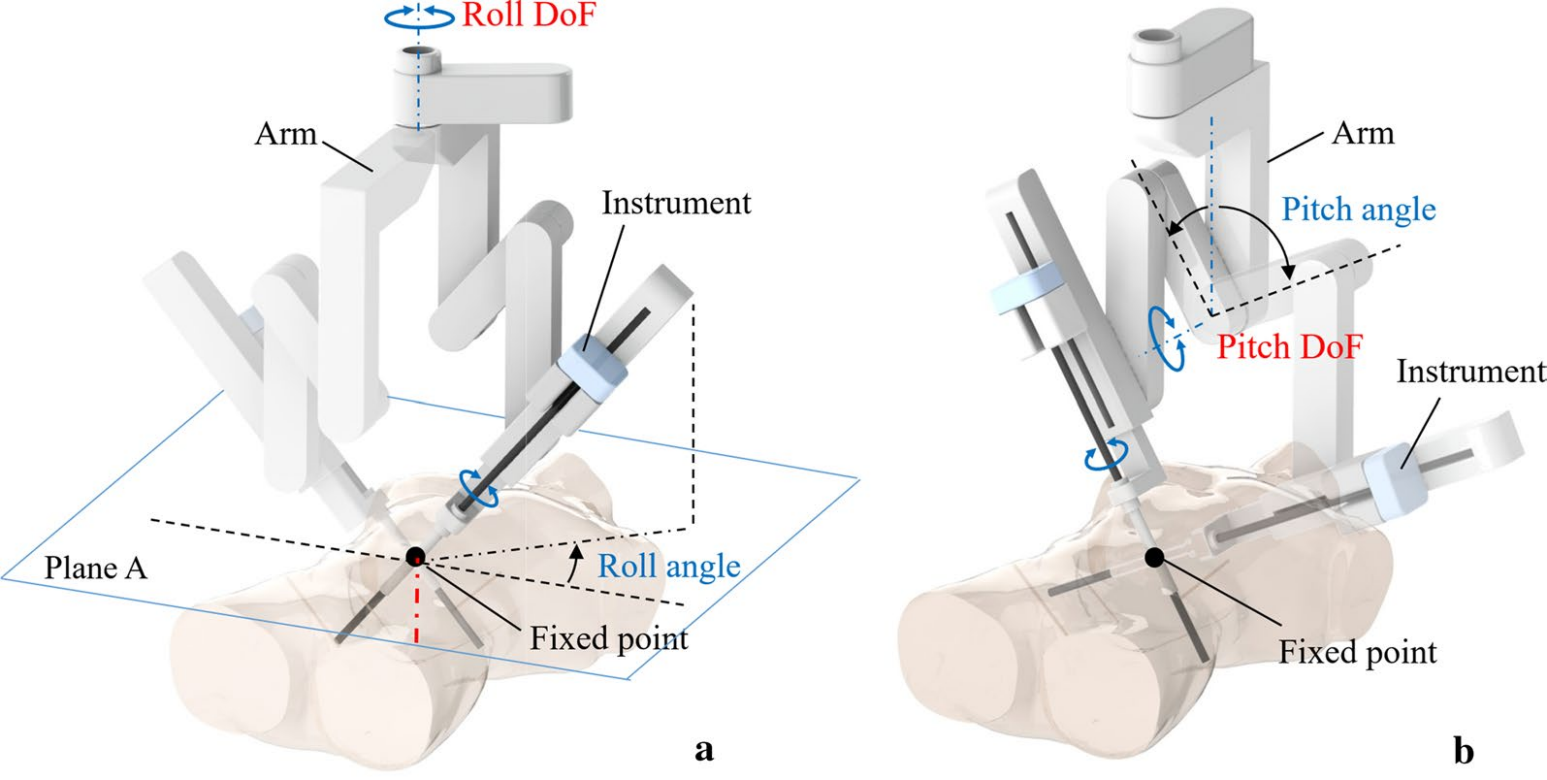
**a** The schematic diagram of fixed point and roll angle. **b** The schematic diagram of the fixed point and pitch angle. The fixed point acts as a fulcrum rather than the incision in the abdomen. The instrument moves around the fixed point in a roll–pitch–roll way. The course of the surgical procedure can be regarded as the combination of roll angle and pitch angle in the tridimensional workspace of the mechanism

The MIISG port setup is more flexible than the LSG setup in spite of the incision point constraint. The DoFs of the laparoscopic rigid instruments are restricted to four, that is, three rotation motions and one translation motion by passing through fixed small incisions [17]. The mechanism of the robot arm part is composed of three active joints and three passive joints. A fixed point has been used to satisfy the incision point constraint by developing an optimized mechanism in the arm part [18]. The fixed point coincides in position with the location of the skin incision leading to positioning algorithms with roll angle and pitch angle, as shown in Fig. 5.

### Surgical technique: MIISG

After general endotracheal anaesthesia was performed successfully, the patient was placed in the supine position with the legs out straight but separated to allow the camera holder to stand between the legs. After the abdomen was sterilely prepared and draped and a foley catheter was inserted, pneumoperitoneum was created with a Veress needle at a point 1 cm superior to the umbilical fossa up to a pressure of 14 mmHg. Then, a 12 mm trocar was inserted for placing a 45° and 12-mm 3D laparoscope as the camera-holder port (A0). The distance between A0 and the umbilicus varied to achieve the proper angle of best visualization. The two ports for the robotic instrument arms (R1 and R2) were set up next. The distances for instrument port placement were measured after insufflation with individualized design, and at least a fist length (10 cm) was maintained between all ports. R1 (8-mm MicroHand cannula) was established at the left midclavicular line superior to the A0 level. R2 (8-mm MicroHand cannula) was placed inside the right midclavicular line superior to the R1 level, with care taken to ensure that the route of the instrument was under the margin of the left liver. In particular, R1 was replaced by a 12-mm trocar as the camera-holder port during the stapling, provisionally. A0 was used as the stapler port, accordingly. In addition, another 5-mm port was placed at the anterior axillary line in the left hypochondrium as an assistant port (A1), which was used to retract the left lobe of the liver, assist stapling and so on.

The patient was placed in the steep reverse Trendelenburg position. The slave robot was brought from one of the available sites on the left side of the patient, and docking was performed in a short time. The camera-holder stood between the patient’s legs. The assistant surgeon was on the left of the operating table. Although the fundus of the stomach was very close to the spine, which is far from the port site, especially for patients with obesity, the range of the instruments was sufficient to reach the fundus field and His angle to isolate tissue and to finish the separation of the fundus.

**Fig. 6:**
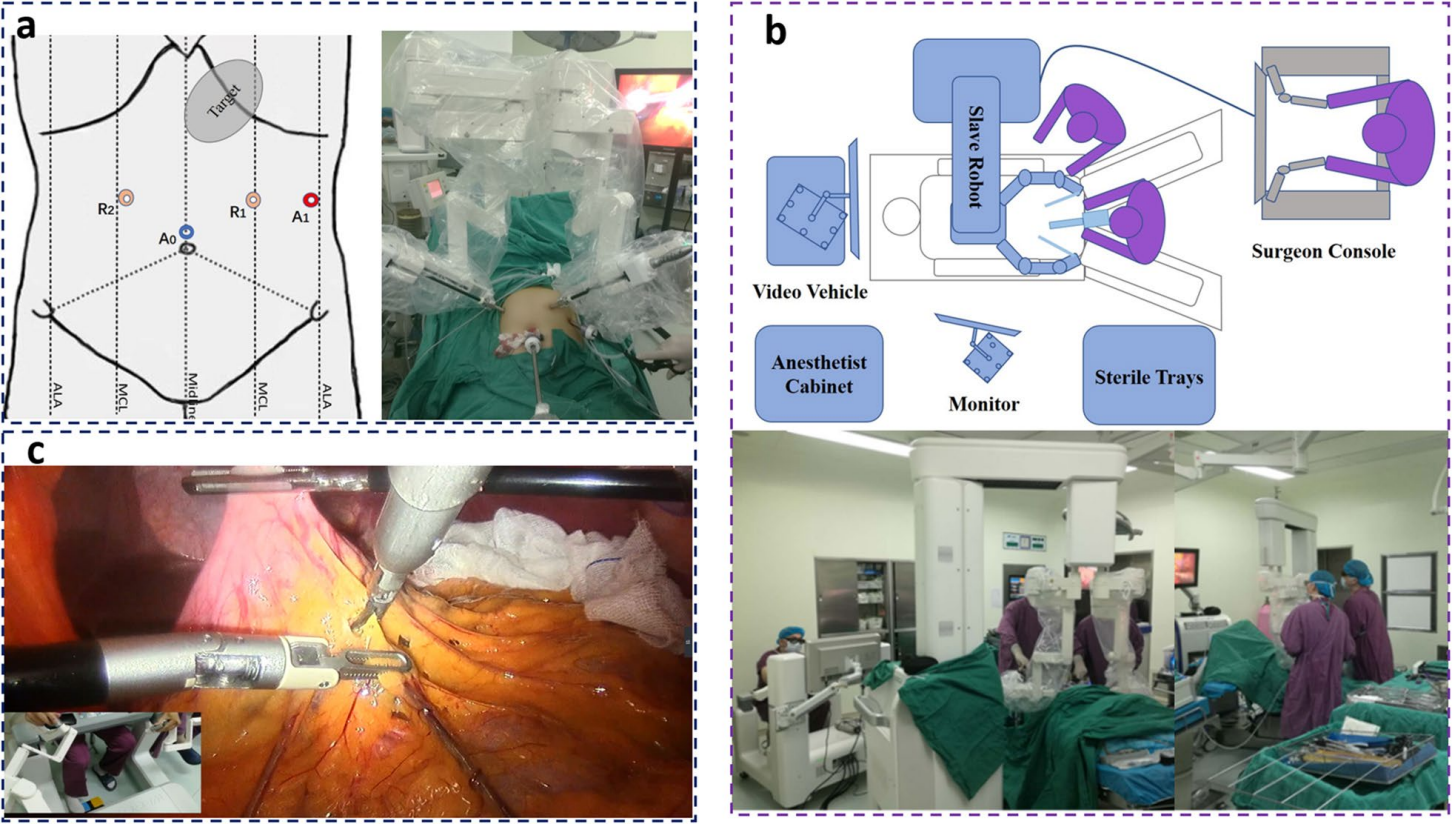
**a** The schematic diagram and real image of port placement, showing the four incision points (R1 and R2 were used for robotic instruments, A0 was used for the laparoscope, and the last A1 was the working channel of a manual instrument, which was used to assist the robot in carrying out the surgery). **b** The schematic diagram and scene of the operating room arrangement during MicroHand SII robot-assisted sleeve gastrectomy (MIISG). **c** Gastrolysis was performed using a MicroHand ultrasonic scalpel

The two main steps of sleeve gastrectomy include the complete dissection and subsequent resection of the greater curvature and gastric fundus. Safe exposure and mobilization of the fundus are regarded especially as the crucial procedure of a sleeve gastrectomy. Gastric mobilization was performed along the greater curvature of the stomach from the prepyloric region, 5 cm from the pylorus, to the His angle using a MicroHand ultrasonic scalpel. The short gastric vessels from the gastrosplenic ligament and posterior gastric adhesions with the pancreas were divided. The left crus was completely defined so that the fundus was adequately mobilized. Instead of a bougie, a gastroscope was placed through the pylorus into the first part of the duodenum and was kept in place to guide the sleeve formation. The console surgeon applied traction on the stomach so that it was splayed out to permit formation of a consistent lesser curvature gastric sleeve. Continuous stapling started 5 cm from the pylorus towards the His angle using a linear stapler through the A0 port to resect the stomach by an adequately trained surgeon standing on the right side of the patient. Instead, R1 was replaced by a 12-mm trocar with the laparoscope inserted. After gastric resection, gastroscopic insufflation of the gastric sleeve was performed after immersing the gastric sleeve with saline infused into the peritoneum in all cases to rule out any leak, bleed, or obstruction. Finally, the resected stomach specimen was removed through the accessory port, and one single-lumen drain was left in the left upper abdomen. The port placement, operation room layout and detailed reality images of the robotic operating procedure are shown in Fig. 6. For continuous data, we present the summary values as median and range, while for blood loss, preop setup time, and percent excess weight loss, we present mean ± standard deviation.

## Results

The patients with obesity included 2 females and 5 males. The age was 35 (20–51) years. The overall operating time was 160 (149–195) minutes, of which preoperative setup time was 28 ± 3 min. The hospital stay was 4 (3–10) days. Intraoperative blood loss was 21 ± 4 ml. No serious complications occurred, such as margin leak, postoperative bleeding or conversion to open. Compared to the baseline, weight [99 (86–136) kg vs 87 (72–101) kg], BMI [32(29.9–49.8) kg/m^2^ vs 27.5 (26.2–37.1) kg/m^2^], and WC [116 (106–140) cm vs 99 (90–122) cm] all significantly improved at 3 months postoperatively (p < 0.05). The percentage excess weight loss (% EWL) at 3 months was 63 ± 10. There were no cases of surgical site infection, gastrointestinal/abdominal bleeding, or conversion to an open operation.

## Discussion

Owing to exciting anthropometric results, SG has currently become the commonest procedure in utilization of bariatric surgery. However, because SG has potential risk of promoting gastroesophageal reflux disease and Barrett's esophagus, some procedures designed to prevent postoperative reflux have also gained a widespread usage [19, 20]. The experience performing SG using the new Micro-hand SII is presented for the first time, but the risk of reflux after MIISG needs to be confirmed by a further large sample study.

In terms of the effectiveness of SG performed by this technique, all of the variables considered, such as BMI change and %EWL, are similar to our laparoscopic data [21]. We have built a standardized technique by summing up our experience. The use of the MicroHand SII robot during the entire intervention is smooth and comfortable. The operating surgeon is seated at the console; it is no longer necessary to force against the resistance of the abdominal wall as with laparoscopic approaches or endure the discomfort of the body position as during use of the daVinci robot. The tremor filtration obtains stable movement despite hand fatigue and minimizes the risk of secondary injury to healthy tissues.

In the da Vinci robot system, the preoperative setup procedure is cumbersome and time-consuming, and interference often occurs between the crowded robotic arms owing to large and heavy passive arms [22]. By comparing those manipulators, the robotic arms of the da Vinci surgical system are more complex and that more passive joints need to be adjusted before surgery compared with the MicroHand robot [23]. With our MicroHand SII robot system, the design of the active slave manipulator combined with the passive arm and swivel head structure can fulfil the requirement of a fast preoperative setup procedure, as there is a quick-exchange interface designed at the end of each manipulator. The function is to realize the quick installation or removal of the surgical tools. Two arms with quick-exchange interface can save setup time. The total operation time is extended in MIISG compared to LSG, by experience, mainly due to the preoperative setup procedure. However, it is far offset by the great advantages of robot-assisted technology especially in complex surgeries. Moreover, the preoperative setup time and the total operation time will be reduced reasonably with more MIISG cases performed [24].

The MicroHand SII surgical robot is a master–slave robot, similar to the da Vinci surgical robot. However, this platform is different from the da Vinci robotic platform in many aspects of the original design. First, da Vinci surgical systems are equipped with four manipulators, including one laparoscope carrier and three working arms. MicroHand SII systems are equipped with only two manipulators to perform surgical procedure and with an assistant holding the laparoscope, similar to conventional laparoscopic surgery. Second, instruments of the da Vinci surgical system are connected to the front end of the retractable manipulators, occupying too much space above the patient. Thus, the workspace reachable is reduced due to intraperitoneal interference between robot arms and other obstacles [22]. However, the two manipulators of MicroHand SII are suspended on the hoisting beam, thus occupying more upper useless space and reserving enough useful workspace for the assistant surgeon, effectively avoiding interference between instruments and surgeons. Third, the DoF arrangement of the instruments in MicroHand SII takes a roll–pitch–distal way which is especially advantageous in the action of stitching. The da Vinci surgical system needs all the joints of the instrument to complete the action of stitching, and the instrument we designed only needs to rotate a joint at the end to complete the action of stitching, which reduces the difficulty of the operation.

The two systems are different in many ways, such as the surgeon operation panel, manipulator design and imaging system. The design of the MicroHand SII arms requires a surgical assistant to hold and maneuver the laparoscopic camera, but this is advantageous because it allows a wider range of the system to adapt to different parts of the endoscopic surgery at the present development stage. A new series design of the MicroHand system is proposed to meet the requirements of more complex operations. A prototype integrated with the slave manipulator carrying a laparoscope and/or a fourth one will be applied clinically soon.

In patients with obesity, the leverage resistance effect is obvious after the trocar passes through the thick abdominal wall [25]. This effect can be effectively mitigated by the MicroHand SII system. In pneumoperitoneum, the abdominal cavity is domed, and the abdominal wall is filled with gas in a uniform radian. Under the MicroHand SII system, the setting of the joint module can further adjust the expanded position and state of the abdominal wall in the presence of pneumoperitoneum, which is conducive to intraoperative trocar regulation. Because the relative positions between the incision point and the slave manipulator are not so strictly determined in the manipulator with three passive joints, the robot can reach different operation positions by adjusting the height of the passive arm and by rotating the swivel head.

## Conclusions

The initial experience showed that the Chinese novel surgical robot system MicroHand SII could be feasibly and safely applied in sleeve gastrectomy. The MicroHand SII robotic platform has obvious advantages in routine use and will be applied widely to more complicated surgical procedures. At the same time, the medical cost of domestic robot-assisted surgery is expected to be reduced by more than half compared to that with the da Vinci robot. Therefore, more patients will benefit from a low-cost and easy-use surgical robot system with the commercialized MicroHand system in future. However, additional studies, including more clinical studies, should be carried out to verify the comprehensive performance of the developed surgical robot.

## Data Availability

The datasets used and/or analysed during the current study available from the corresponding author on reasonable request.

## Abbreviations

LSG: Laparoscopic sleeve gastrectomy
IFSO: The International Federation for the Surgery of Obesity and Metabolic Disorders
DoF: Degrees of freedom
3D: Tridimensional
MBSAQIP: The Metabolic and Bariatric Surgery Accreditation and Quality Improvement Program
CSMBS: The Chinese Society for Metabolic & Bariatric Surgery
MIISG: MicroHand SII robot-assisted sleeve gastrectomy
BMI: Body mass index
WC: Waist circumference
% EWL: Percentage excess weight loss
